# Supplementary material for: Toward improvement of knowledge of financial conflicts of interest in a large medical school in France
Source: PLoS One. 2023 May 22;18(5):e0285894. doi: 10.1371/journal.pone.0285894 (PMC10202289; doi:10.1371/journal.pone.0285894)
Supplement: S1 Appendix — (DOCX) [file pone.0285894.s001.docx]

**Appendix: Summary of the charter**

Faculties are committed to scientific integrity

They commit to respect the proposals for the implementation of the national charter of ethics for the research profession.

The Faculties are committed to ethics

The Faculties are committed to respecting and respect the rules of ethics and integrity and integrity recommended by all the professional of the professions of which they are composed. The Faculties are committed to the general of the promotion of gender equality and the equality between men and women and the fight against discrimination of any kind, and of the accessibility of the handicap.

Ethical and deontological training for responsible conduct

Mandatory ethics training of ethics and deontology are reinforced within the Faculties.

Transparency and declaration of links of interest

Faculty must disclose students of their links of interest as a preamble to the courses they teach, whatever their teaching, regardless of its nature (written, oral, online).

Benefits and gifts

Gifts, even of small value, funded by the industry are not permitted.

Good teaching practices in relations with the health industries

The health products mentioned in the course are necessarily in the form of an international non-proprietary name (INN), without any promotion. The same applies to for medical devices. The students have the right to exercise a duty of confidentiality when they are in a position of influence. They may express themselves freely without exposing themselves to grievance or sanctions from their superiors.

Links with care partners

Exposure to the risk of conflict of interest in the care sectors must be prevented. Representatives of the pharmaceutical and health product industries are not allowed to meet with academic staff in health care areas or or in the presence of students. This means that they cannot organize presentations in the presence of students

Non-compliance with the charter

The present charter is opposable to all actors within the Faculties, regardless of their rank, status, or hierarchical level. In the event of a documented suspicion of of reprehensible conduct, the students are encouraged to contact the Ethics Commission
