# Supplementary material for: Toward improvement of knowledge of financial conflicts of interest in a large medical school in France
Source: PLoS One. 2023 May 22;18(5):e0285894. doi: 10.1371/journal.pone.0285894 (PMC10202289; doi:10.1371/journal.pone.0285894)
Supplement: Attachment — Submitted filename: response to reviewers 14-04-23.docx [file pone.0285894.s002.docx]

We thank the Reviewer for his positive appreciation of our work and for his helpful suggestions that have all been followed in the manuscript.

*Throughout the manuscript, the use of the term "charter" instead of "chart" should be harmonized: there are still quite a few passages where the word "chart" is used*.

Done

*Page 7: "This questionnaire was also sent to the Conference of the Deans who did not answer it..." ->: I think that the use of ellipsis is not very common in scientific articles.*

We agree. We have suppressed the suspension points and slightly changed the sentence.

*Page 7-8: In the paragraph "The accuracy of results of previous studies is beyond doubt... his amendment occurred after our survey was conducted." -> to me, here there are two different ideas: the limitations of the Formindep study and the tools put in place to limit COI. The paragraph should be split in two in order to maintain coherence.*

We have followed the Reviewer’s suggestion and modified the paragraph accordingly.

*Page 9: "Last, we did not investigate the behavior of the teachers, but they probably share responsibility for the poor results with the students." -> I don't understand what you mean here. From the turn of phrase, it is understood that the students would share some responsibility for the poor results of the study. I think that needs to be clarified because the responsibility for the low knowledge of the charter does not lie with them at all.*

The Reviewer is perfectly right. We have modified the unfortunate sentence.

*Thank you for your attention to my comments and congratulations on the great work done on this article*

Many thanks again for your very insightful review.
